# Enhancing Spinach Growth and Soil Edaphic Factors Using Aquatic Weed–Derived Biochar

**DOI:** 10.1155/sci5/9303188

**Published:** 2025-09-25

**Authors:** Muntaha Munir, Aisha Nazir

**Affiliations:** Institute of Botany, University of the Punjab, Lahore 54590, Pakistan

**Keywords:** aquatic weeds, biochar, pyrolysis temperature, soil amelioration, valorization

## Abstract

*Phragmites australis* (Cav.) Trin. ex Steud and *Lemna minor* L. are non-native aquatic weeds. They disturb the habitat dynamics by competing with native flora for water and nutrition, deplete oxygen in water bodies, destroy water quality, and create habitat for mosquitoes and other parasites. Valorization of this huge biomass into biochar is a sustainable approach to address both environmental and agricultural challenges. It not only mitigates the climate issues by proper management but also sequesters carbon and improves soil quality. The current study manifests the utility of *Phragmites australis* and *Lemna minor* biomass as a feedstock for pyrolysis, at 400°C, 500°C, and 600°C, to design *Lemna minor* biochar (LMBC400, LMBC500, and LMBC600) and *Phragmites australis biochar* (PABC400, PABC500, and PABC600). These biochars were added as a soil conditioner to estimate the productivity of test crops. The results demonstrate that ash content, pH, electrical conductivity (ECe), and fixed carbon are directly proportional to pyrolysis temperature, whereas oxygen, hydrogen, nitrogen, volatile contents, and bulk density (BD) are inversely proportional to pyrolysis temperature. Scanning electron microscopy (SEM) and Fourier transform infrared (FTIR) spectroscopy analyses proved that high pyrolysis temperature increases the porosity and phenolic compounds in biochar, which improves the surface quality. The percentage of nutrients such as Mg, Fe, N, Ca, N, P, K, and Zn increases by elevating pyrolysis temperature due to unlocking and release under the influence of heat. Soil quality parameters (viz. pH, BD, ECe, water holding capacity [WHC], total dissolved solids [TDS], and soil organic matter [SOC]) improved in favor of plant growth conditions, so the yield of test crops increased. So, LMBC600 and PABC600 had great potential to improve soil and productivity. It is a promising approach to manage this colossal volume of *P. australis* and *L. minor* through green technology by recycling this biowaste into a worthy product biochar, which is an alternative to chemical fertilizer in agronomical practices, which leave eco-toxic footprints and play havoc to the environment.

## 1. Introduction

Population explosion and rapid loss of soil fertility impart a serious dilemma to farming practice and food security as well as environmental pollution [1, 2]. Use of commercial fertilizer (CF) in conventional agricultural practices proves ecologically toxic [3, 4], leading to deterioration of water and soil with excessive nutrient loads, depletion of soil organic matter (SOC), and the decrease in soil fertility, along with deformation of soil structure, composition, texture, and low water-holding capacity (WHC). These phenomena lead to low soil fertility, failure of cropping systems, and reduced yields [5]. On an average, 2%–10% of nutrients of CFs leach into water tables or other water channels, and the amount of water may vary conditionally. This leached water having nutrients leads to eutrophication, so it is not much beneficial for plants [6, 7]. Most of the primary nutrients (NPK) were inaccessible for crop uptake. The drastic effects of synthetic CF can restrict plants' growth and yield. Jayachandran and Rao [8] explained that industrialization, poor agricultural practice, and other anthropogenic activities lead to eutrophication of water bodies, where exotic aquatic weeds prevail, disturb the ecosystem globally, and alter the species composition [9]. Aquatic weeds cause anoxic conditions, provide habitat to mosquitoes and other parasites, emit methane due to the accumulation of methanogenic bacteria in ponds, deteriorate the water quality, hinder fisheries, block the waterways, disturb the aquatic system, and destroy the aesthetics of recreational lakes. [10].

*Phragmites australis* (Cav.) Trin. ex Steud. (common reed) is an emergent, perennial weed. It has cane-like stems on a rhizome. It grows along water marshes, drainage canals, ditches, and edges of shallow lakes and sandy banks. Stems may reach 5-6 m in height, 4–10 mm in diameter, and 10–25 cm internodes. Enormous aerenchymatous tissues provide oxygen in the rhizome. Roots extend from rhizomes. Leaves appear alternate, narrow, tapered, and lanceolate (20–70 × 1–5 cm). The inflorescence makes a panicle (dull purple to yellow). Chloroplast DNA sequencing has proved that *P. australis* is native to North America [11]. *Lemna minor* L. (duckweed) is a common aquatic weed consisting of an undifferentiated shoot. It has basal and nodal portions only; secondary roots made up of hypodermal cells are present having a temporary root sheath; flowers are rarely formed and are reproduced by buds [12]. *Lemna minor* natively belongs to North and South America. Duckweed is popular for accumulating phosphorus (P).

To promote sustainable management of these noxious weeds, ecosystem stability, and soil management, it is mandatory to manage aquatic biomass and maintain enough organic matter (OM). Organic fertilizers (mulches, composts, manures etc.) can upgrade the soil quality [13–15]. Although, these amendments of soil work transiently due to rapid purification [16]. Moreover, these organic amendments can lead to organic and inorganic pollution (heavy metals, microplastics, and persistent organic pollutants) in soil [17, 18].

To compensate for such dilemmas, manufacturing and utilization of “biochar” that has analogous conditioning effects on soil rather than other amendments are considered [19–21]. “Biochar” is a carbonaceous, lightweight, and porous material obtained by the pyrolysis of biowaste at various temperatures in the absence of oxygen [22]. Biochar remains in soil for the long run due to its high resistance to decomposition [23]. Biochar is becoming popular as a soil conditioner due to its budget-friendly and green biotechnological features, which ameliorate soil and sequester carbon. Biochar ameliorates the soil's chemical and physical properties, with a significant enhancement in the yield and productivity of the crop. The amendment of biochar into soil leads to the availability and retention of nutrients, and water in the soil increases [24, 25], aggregation and porosity of soil increase [26, 27], and the uptake of nutrients boosts up [28, 29]. Biochar carries nutrients and releases them slowly according to the plant's needs, so it prevents the offsite loss [30, 31].

“Biochar” is an emerging and modern green biotechnological product; it has reflected remarkable effects on the yield of crops and nutrient value [32–34]. There is still a gap in research about the toxicity and fate of biochar in the ecosystem, so this research aims to encompass the present gap by exploring the effect of common reed and duckweed biochar on soil conditioning or amelioration and productivity of *Spinacia oleracea* L. in the biochar-mixed soils. It was hypothecated that the addition of biochar would significantly uplift the soil physicochemical properties and productivity, presenting a hardback correlation between biochar and soil-amelioration setup.

This research encompasses a novel approach by emphasizing the utilization of abundant aquatic weed biomass to produce biochar. These aquatic weeds have been neglected as waste, but they act as a bioresource. Previous studies supported biochar made from agricultural and forestry waste, but the current research examines the potential of aquatic weed biochar as a soil conditioner. Moreover, our study explored the specificity of aquatic weeds' biochar to enhance soil fertility and plant growth, which offers a novelty. This innovation extends the scope of research on biochar and provides a sustainable solution to manage invasive aquatic weed and soil conditioning. Biomass harvesting of aquatic weeds does not create any environmental or ethical issues. A preliminary draft of this research was presented as an abstract at the 9th International and 18th National Conference of Plant Scientists (INCPS-2024), Bahauddin Zakariya University, Multan, Pakistan [35]. In terms of the environment, the practice will not lead to deforestation, loss of biodiversity, and soil misuse, which means that the research will not harm the ecosystem in any way. Ethically and socially, the process does not negatively affect local communities but provides a viable mechanism for handling menacing submerged weeds. This would be a good way of dealing with invasive species besides encouraging a healthy ecosystem without any negative effects.

The current research was conducted under the following objectives: (1) To evaluate the physical and chemical characteristics of biochar, (2) to estimate the effect of biochar treatment on soil edaphic characteristics, and (3) to evaluate the effect of biochar-amended soil on crop (spinach) productivity.

## 2. Methodology

### 2.1. Survey, Feedstock Collection, and Preparation

**Figure 1:**
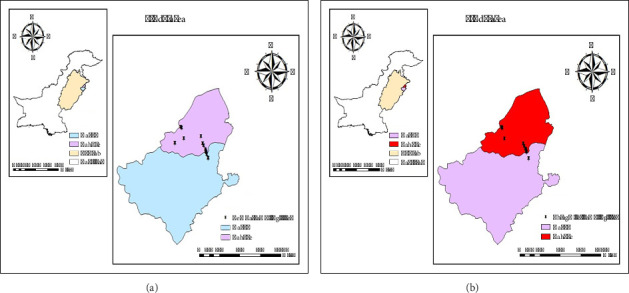
(a) Sampling sites of *Lemna minor* (LM). (b) Sampling sites of *Phragmites australis* (PA).

*Phragmites australis* (Cav.) Trin. ex Steud., the common reed, and *Lemna minor* L., the duckweed, were collected from June 1 to 30th July, 2023, from different waterways of Kasur and Lahore, Punjab, Pakistan. These sites were selected based on a huge infestation of aquatic biomass over the water surface.  Figure 1 presents the sampling sites. The collected biomass (5 kg in triplicate) was washed with tap water to remove all debris and air-dried in direct sunlight for 15 days. Long stems of *P. australis* were crushed into 1 cm chips with the help of an electric chopper, and then oven-dried throughout the night at 70°C to attain a constant weight and volatile loss of moisture.

### 2.2. Biochar Production

Dried and crushed PA and LM biomasses were pyrolyzed at 400°C, 500°C, and 600°C (slow pyrolysis). Slow pyrolysis is a low heating rate thermal decomposition process in which biomass is heated with little or no oxygen present at long residence times. The process is usually conducted between 350°C and 600°C and can vary in time between a few minutes to hours. Slow pyrolysis produces biochar as the primary product. Slow pyrolysis has a longer heating and residence time, which makes it more favorable to carbon retention in the solid phase instead of maximum liquid or gas yields. Such an approach is common when it comes to the production of biochar that is aimed at soil restoration, carbon storage, and environmental treatments. Heating rate means the speed at which temperature is increased during pyrolysis; it will influence whether the process is more biochar (slow pyrolysis) or liquid/gas-yield-oriented (fast/flash pyrolysis). These pyrolysis temperatures were selected to investigate the best temperature to obtain perfect qualities of biochar having high carbon [36, 37].

Abbreviations were assigned to the biochar samples based on temperature and the nature of the feedstock. *Phragmites australis* biochar (PABC) 400, PABC500, and PABC600 represent biochar produced by pyrolyzing *P. australis* biomass at 400°C, 500°C, and 600°C. Similarly, *Lemna minor* biochar (LMBC) 400, LMBC500, and LMBC600 indicate the biochar produced at 400°C, 500°C, and 600°C. Slow pyrolysis was performed in a locally designed semiautomatic charcolator, made up of two cylindrical combusting containers, which fit into the cemented metal alloy furnace. The container tubes had a diameter of 2 feet and a length of 2.5 feet, with a capacity of 5 kg of feedstock. The furnace was connected to an outer pipe and chimney to release gases and vapors. One digital thermostat was placed near the base of the burning chamber to monitor temperature. The residence time varied depending on the type of feedstock, and after a few hours, the required temperatures were attained [38]. The furnace was opened after cooling down, and chars were collected, weighed, ground, sieved, and packed in polythene bags and stored in a desiccator to avoid moisture (for detailed analysis and usage). All pyrolysis experiments were carried out in accordance with standard laboratory safety precautions such as the wearing of personal protective equipment (gloves, lab coat, and safety glasses), adequate ventilation to prevent gas build-up, and ensuring that the furnace was completely cooled before it was touched.

### 2.3. Analysis of Biochar

#### 2.3.1. Yield of Biochar Samples

The theoretical yield of prepared biochar samples was estimated by following the formula as used by Das et al. [39]:

(1) $$\begin{array}{c} \text{theoretical yield }\left(\%\right)=\frac{\text{mass of biochar after pyrolysis }\left(g\right)}{\text{mass of feedstock before pyrolysis }\left(g\right)}\times 100, \end{array}$$

where feedstock means the raw material or biomass of aquatic weeds, which were subjected to pyrolysis (three batches of each feedstock were used for one round of pyrolysis [*n* = 3]).

#### 2.3.2. Proximate Analysis of Biochar Samples

The ash content (AC%) of biochar samples was ascertained by calculating the ratio of residual masses after combustion at 500°C–600°C/1 h in a muffle furnace, and the OM% was determined by subtraction. Volatile OM (VOM%) was computed by loss of ignition rate; the fixed carbon content (FC%) was also determined according to ASTM International standards [40].

(2) $$\begin{array}{c} \text{Moisture content }\left(\text{MC}\right)\;\left(\%\right)=\frac{\text{air}‐\text{dried BC mass}–\text{BC dried at }105^{{}^{\circ}}C\text{ mass}}{\text{air dried BC mass}},\\ \text{ Volatile carbon }\left(\text{VC}\right)\;\left(\%\right)=\frac{\text{air}‐\text{dried BC mass}–\text{BC dried at }950^{{}^{\circ}}C\text{ mass}}{\text{air}‐\text{dried BC mass}},\\ \text{AC }\left(\%\right)=\frac{\text{BC dried at }950^{{}^{\circ}}C\text{ mass}–\text{BC dried at }750^{{}^{\circ}}C}{\text{BC dried at }950^{{}^{\circ}}C\text{ mass}},\\ \text{FC }\left(\%\right)=100-\text{MC}\%+\text{VC}\%+\text{AC}\%. \end{array}$$

The higher heating value (HVV) was ascertained by following the formula provided by Das et al. [39]:

(3) $$\begin{array}{c} \text{HVV}=\left(0.0078\text{ ash}\right)-\left(0.1559\text{ VM}\right)+\left(0.3536\text{ FC}\right), \end{array}$$

where FC indicates fixed carbon and VM indicates volatile content. All values were taken three times to maintain accuracy.

#### 2.3.3. Ultimate Analyses of Biochar Samples

Elemental analysis of the biochar samples was carried out on an elemental analyzer (Thermo Fisher, MA, USA) to determine their elemental composition, C, nitrogen [N], H, S, and O (*n* = 3). First, the samples were ground and then homogenized before being combusted in the high-temperature analyzer. The gas emissions in the process of combustion were identified and measured to calculate the proportions of C, N, H, and S. The content of oxygen was obtained by comparing the total mass of the sample and the sum of the amount of the C, N, H, and S. The measurements were handled with calibration and quality control (Cont) processes, and standards were used and as well as replicates to ensure accuracy and reliability.

#### 2.3.4. Physicochemical Analysis of Biochar Samples

To conduct the physicochemical content of the biochar samples, the electrical conductivity (ECe) and pH were obtained by adding 1 g of finely ground biochar in 20 mL of distilled water (DW) and shaking the mixture vigorously for 3 h on an orbital shaker at 25°C–28°C room temperature. The solution was filtered through Warman filter paper 42 (2.5 μm) to conduct analyses by the procedure described by Bordoloi et al. [41] after shaking well. ECe of the filtrate was determined in the unit of micro-Siemens per meter (μS/cm) by an ECe meter, and the pH was measured by a multimeter, as mentioned, in Section 2.4. The bulk density (BD) (BD g/cm^3^) of the powdered biochar was determined by adding the powder content to a 100 mL beaker, gently tapping 3 times until the contents were packed securely, and measuring the packed volume of the biochar in the glass cylinder by using the following formula:

(4) $$\begin{array}{c} \text{BD}=\frac{\text{weight of biochar in the powder}\left(g\right)}{\text{volume of biochar in the glass cylinder}\left({\text{cm}}^{3}\right)}. \end{array}$$

The cation exchange capacity (CEC) assessed was based on a solution of ammonium acetate (unit: cmol(c)/kg), respectively, following the instructions of Chapman [42]. In short, 1 g of biochar was shaken with 50 mL of ammonium acetate solution (1 M) throughout a period of 2 h to replace with 1M KCl solution. Measurement of the displaced ammonium ions was carried out immediately after filtration, and CEC was calculated by the flame photometer (Sherwood Scientific, Model 410, UK). To analyze nutrients, 1 g biochar sample was digested using 4 : 1 ratio of perchloric acid (HClO_4_) and nitric acid (HNO_3_) and placed on a hot plate until a clear solution was obtained. The last volume was brought to 100 mL with DW, and the nutrients were analyzed (units; mg/kg) as in Baird et al. [43]. Each measurement was recorded in triplicate to ensure accuracy and reproducibility.

#### 2.3.5. X-Ray Diffraction (XRD) Pattern of Biochar Samples

The XRD technique was employed to determine the crystal components of biochar samples. The XRD pattern of the biochar samples was obtained with an automatic diffractometer (model PW 1710 diffractometer, PW 1729 X-ray generator). The crystalline index of biochar was speculated. Spacing of planes of atoms in the crystal lattice was estimated and recorded in the form of peaks of the XRD pattern according to Bragg's law [44].

(5) $$\begin{array}{c} N\lambda =2d\times \sin\;\theta , \end{array}$$

where *N* is the order of reflection, λ is the X-ray's wavelength, *d* is the distance between diffracting planes in the crystal, and *θ* indicates the angle between the X-ray beam and the diffracting plate.

#### 2.3.6. Nutrient Analysis of Biochar Samples

To estimate the minerals/nutrients (magnesium [Mg], N, phosphorous [P], potassium [K], calcium [Ca], iron [Fe], and manganese [Mn]) in biochar samples, PABC and LMBC biochar samples were ground, sieved, weighed to 1 gm, and put into 250 mL beakers. 5 mL concentrated HNO_3_ was added and covered with aluminum foil and left for 12 h. These samples were again heated on a hot plate; some drops of HCl were added until the solution became transparent and clear. The volume of solutions rose to 100 mL by adding DW to make it ready for analysis by different analyzers.

The total P (TP) was calculated by a spectrophotometer (BioTek, Epoch 2, USA) at the wavelength of 880 nm. Total K and N were ascertained through a flame photometer as measured by Wogi et al. [45]. Minerals were quantified on atomic absorption spectroscopy (AAS) by standard protocol as utilized by Enders et al. [46]. Estimation of water-soluble mineral elements (Fe, Ca, Mg, zinc [Zn], Mn, and copper [Cu]) present in PABC and LMBC samples was performed by the procedure as elaborated by Bian et al. [47]. All measurements were recorded in triplicate with a precision of ±0.1%.

#### 2.3.7. Surface Morphology, Composition, and Functional Groups Analyses of Biochar Samples

Fourier transform infrared (FTIR) spectroscopy of biochar samples was analyzed and functional groups of biochars were estimated by using the spectrophotometer. At the range of 450–4500 cm^−1^, these spectra were obtained to expose various functional groups or aromaticity present in PABC and LMBC. The surface morphology was ascertained by scanning electron microscopy (SEM) by SEM-Energy system (JEOL JSM-6360LV).

### 2.4. Soil Analysis

Soil from each pot was collected, air-dried, hand-crushed, and sieved using a 2-mm·mesh sieve to eliminate bigger pebbles and contaminants. The soil saturation extract with DW was prepared at the ratio of 1 : 10 (w [weight]/v [volume]), filtered through Whatman filter paper 42 (2.5 μm) to perform physicochemical analyses. Estimation of pH and total dissolved solids (TDS) was measured as parts per million (ppm), and ECe was measured as deciSiemens per meter (dS/m) with the help of a precalibrated portable digital multimeter (Hanna Instruments HI 9811-5). The values of OM were measured as a loss on ignition (LOI), and total organic carbon (TOC) (g/kg) was measured using the Walkley–Black method. BD (g/cm^3^) was determined in grams per cubic centimeter (g/cm^3^), and CEC (cmol(c)/kg) was calculated according to the procedure developed by Chapman [42]. The mineral content of all soil amendments was analyzed by atomic absorption spectrophotometry (AAS) and followed by acid digestion as reported by Yarrakula [48]. The value of WHC in the soil was calculated as that of Wogi et al. [45]. Soil samples were also analyzed prior to sowing and after harvest to determine the effects of soil amendments on the characteristics of soils (number of replicates of each parameter = 3).

Certified reference materials and standard methods were used in calibration and validation. Proximate and ultimate analyses were determined as per the ASTM standards. CHNS instrument calibration was performed with acetanilide as a calibration substance. Multielement nutrient tests (e.g., K, Ca, Mg, Na, Fe, and Zn) were calibrated using multielement standard solutions traceable to the National Institute of Standards and Technology (NIST). Physicochemical characteristics such as pH, EC, and others of soil and biochar were measured according to ISO standards to allow exact and reproducible results.

### 2.5. Experimental Setup

The experiment was managed and conducted according to a randomized complete block design (RCBD) to reduce possible errors (experimental units were arranged in blocks in which treatments were assigned in a randomized way). Each treatment was performed in three replicates. Pots having the capacity of 2 kg of soil (loamy soil, pH = 6–7.5) were used. The experimental setup consisted of the following treatments: (1) Cont soil (without any biochar or fertilizer), (2) PABC amendment in soil at three levels (2%, 3%, and 5% w/w [weight/weight]), (3) LMBC amendment in soil at three concentrations (2%, 3%, and 5% w/w), (4) PABC and LMBC (mixture 1:1) amendment in soil at three concentrations (2%, 3%, and 5% w/w), (5) amendment with CF (Repsol NPK 20 : 20 : 20 [2 g/1 kg soil in pot] for spinach).

All the treatments were applied to the soil before sowing seeds, and pots were kept in a greenhouse for 2 weeks to promote the action of biochar in the soil. The recorded temperature range of the greenhouse was 25°C–28°C, and humidity was 55%–73%, along with natural light for 12–14 h per day with natural air circulation to avoid fungal diseases and uniform distribution of humidity and temperature. In this experiment, the spinach was selected as a test plant because of its fast growth and life cycle. Three healthy seeds were sown in each pot. Pots were watered and monitored daily during the growth period. The plants (triplicates) were continuously grown for 50 days to get maximum yield and maturity.

### 2.6. Vegetable Productivity

#### 2.6.1. Plant Height and Root Length (cm)

The plant height (cm) of a mature plant was calculated by using a measuring scale. The length was measured from the base to the top of the plant along the stem. Similarly, the root length (cm) was estimated after washing the roots with tap water.

#### 2.6.2. Number of Fruits per Plant

At full growth, the fruits were picked from each plant, counted, and packed in a zipper polythene bag.

#### 2.6.3. Fresh and Dry Weight (%)

To determine the fresh weight, spinach plants were harvested from each treatment unit and were weighed on an electric weight balance to get Wt1. After that, these samples were kept in an oven at 70°C for 48 h. Dry weight (Wt2) was determined by an electric weight balance after drying in an oven. To calculate dry matter (%), the following formula was applied as given by Garnier et al. [49]:

(6) $$\begin{array}{c} \text{dry matter }\left(\%\right)=\frac{\text{Wt}1}{\text{Wt}2}\times 100. \end{array}$$

#### 2.6.4. Crude Protein (%)

Crude protein (%) was estimated by the Kjeldahl apparatus. The N of protein was converted into ammonium sulfate by acid digestion with conc. H_2_SO_4._ This digested solution was distilled with boric acid (H_3_BO_3_) to collect ammonia. The titration of the solution was performed against 0.01 N H_2_SO_4_. The crude protein (%) was calculated by the following formula as given by Casal et al. [50]:

(7) $$\begin{array}{c} N_{2}\%=\frac{\text{volume of }H_{2}{\text{SO}}_{4}\left(\text{mL}\right)\times \text{acid }N_{2}\times 0.014}{\text{weight of the sample}\left(\text{gm}\right)}\times 100,\\ \text{crude protein}\left(\%\right)=N_{2}\times 6.25. \end{array}$$

#### 2.6.5. Crude Fat (%)

Crude fat was ascertained by the method of ether extraction by using a Soxhlet extraction apparatus. 20 g of dried and ground sample were placed in a crucible. The crucible was fitted below the condenser. Petroleum ether (40–45 mL) was poured into a beaker, which was then kept above the condenser, and the water supply was switched on for 24 h. The sample was picked after 24 h, and extracts of ether were collected and measured. The extract was evaporated in an oven as described by Garces and Mancha [51].

(8) $$\begin{array}{c} \text{Crude fat }\left(\%\right)=\frac{\text{wt}.\text{ of dried ether extract}}{\text{wt}.\text{ of the vegetable sample}}\times 100. \end{array}$$

#### 2.6.6. Crude Fiber (%)

One gram of dried and ground sample of vegetable was taken in a conical flask, and 200 mL of H_2_SO_4_ was added and kept for 30 min, followed by acid digestion, and then subjected to base digestion with 200 mL NaOH for 30 min and washed with water and acetone. The rest of the residual mass was kept in a preweighed crucible and then kept in an oven for 8 h at 60°C. After drying, the sample was kept in a muffle furnace at 600°C for 12 h. The weight of the ashes sample was calculated. To find out the crude fiber %, the following formula was applied as used by Van Soest and McQueen [52]:

(9) $$\begin{array}{c} \text{crude fiber }\left(\%\right)=\frac{\text{wt}.\text{ of ash}}{\text{wt}.\text{ of raw vegetable sample}}\times 100. \end{array}$$

### 2.7. Statistical Interpretation of Data

The collected data were analyzed carefully by using a two-way analysis of variance (ANOVA) to estimate significant differences between different treatments (*n* = 3). Main effects and interactions between various factors were studied by this statistical approach. Fisher's least significant difference (LSD) test was applied to further explore the differences. It is a post hoc test employed to compare the treatment's mean values. OriginPro (Version 8.1) was used to conduct these analyses with *p* values set as <0.05, and the correlation between biochar properties, soil properties, and plant growth was analyzed by Pearson's correlation matrix for statistical significance.

## 3. Results

### 3.1. Yield of Biochar Samples

**Figure 2:**
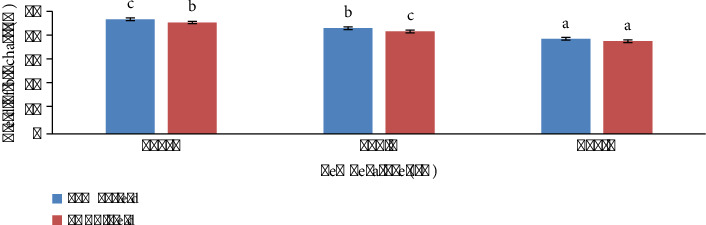
Biochar sample yield (%).

The amount of yield is correlated with pyrolysis temperature. In this study, it is obvious from Figure 2 that the yield decreases antagonistic to elevating temperature. LMBC yield was found to be 47% ± 0.57, 43% ± 0.60, and 38% ± 0.57 at 400°C, 500°C, and 600°C. PABC yield was found to be 45.6% ± 0.7, 42% ± 1, and 38% ± 1 at 400°C, 500°C, and 600°C (*n* = 3, *p* value <0.05). On average, LMBC yield decreased by 19% from 400°C to 600°C, whereas PABC yield was reduced by 16% between the same pyrolysis temperatures.

### 3.2. Proximate Analysis of Biochar Samples

**Figure 3:**
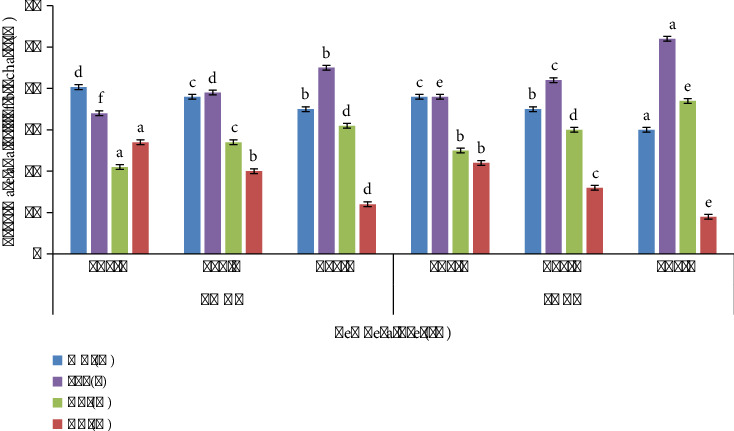
Proximate analysis of biochar samples.

Pyrolysis temperature greatly affects the physicochemical properties of biochar (Figure 3) by using *p* value of <0.05 as statistical significance (*n* = 3). In the current study, among all treatments, the lowest MC was seen in LMBC and PABC biochar samples made at 600°C, i.e., 35% and 30%, respectively. The MC of LMBC was high compared to that of PABC. However, the MC decreases by increasing the pyrolysis temperature. By increasing the temperature, the volatile matter decreases, as shown in the graph, where 600°C produced biochar samples with minimum volatile matter as compared to 400°C and 500°C. PABC at 600°C revealed the lowest amount of volatile matter. LMBC had the lowest VC at 600°C, i.e., 12% and PABC had 9% VC at the same temperature.

### 3.3. Ultimate Analysis of Biochar Samples

**Figure 4:**
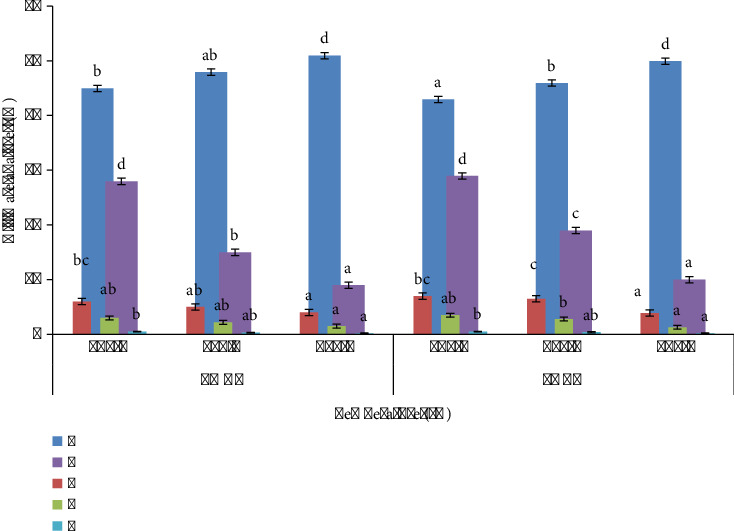
Ultimate analyses of biochar samples.

The prepared biochar samples were subjected to ultimate analyses, with mean determination of C, H, N, O, and S (values are given in average of triplicates, and statistical significance was estimated using *p* value as <0.05). C/N ratio indicates the stability of BCs and their capacity for nutrient retention. Among the biochar samples, the C content was higher at 600°C (51% in LMBC and 50% in PABC). H content decreased with respect to increasing pyrolysis temperature (4% in LMBC and 3.9% in PABC). N decreases when the pyrolysis temperature increases (1.5% in LMBC and 1.3% PABC). O also decreases by enhancing the pyrolysis temperature as depicted in Figure 4. The quantity of sulfur decreases with respect to rising pyrolysis temperature (Figure 4).

### 3.4. Physical and Chemical Analyses of Biochar Samples

**Figure 5:**
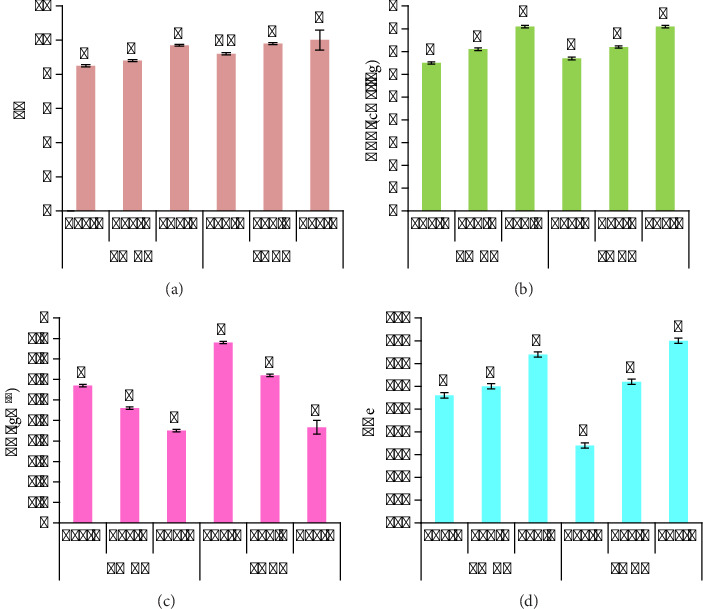
Physicochemical analysis of biochars. (a) pH. (b) CEC (cmol/kg). (c) BD (g/cm^3^). (d) ECe (μs/cm).

The pyrolysis temp affects pH in a significant way (*p* value <0.05). As shown in Figure 5 the pH of biochar rushes toward alkaline by elevating the temperature of pyrolysis. PABC revealed the highest pH, i.e., 10, as compared to LMBC, i.e., 9.7, at 600°C. CEC of the biochar was significantly affected by pyrolysis temperature (*p* value <0.05). CEC of both biochars was the same, 8.1 cmol(c)/kg, at 600°C. The BD of both biochars significantly (*p* value <0.05) reduced with increasing pyrolysis temperature. Maximum reduction of BD was seen at 600°C, i.e., 0.45 g/cm^3^ of LMBC and 0.46 g/cm^3^ of PABC. ECe of biochar enhances with respect to pyrolysis temperature due to greater carbonization, movement of electrons, doping effect, and composition of polymers formed after pyrolysis. The highest ECe was reflected by PABC, i.e., 460 μS/cm at 600°C (*p* value <0.05).

### 3.5. Nutrient Analysis of Biochar Samples

**Figure 6:**
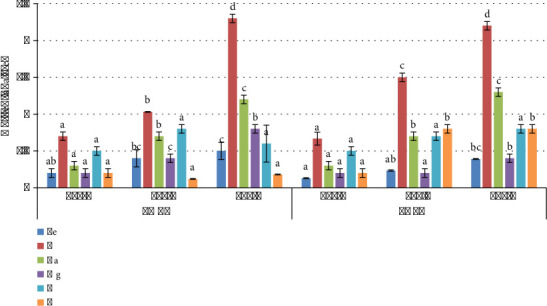
Nutrient analysis of biochar samples.

The pyrolysis temperature greatly influences the nutrient ratio and characteristics of biochar, as depicted in Table 1. LMBC revealed that the Fe content increases up to 0.5% at 600°C, and LMBC had 0.39% Fe, when formed at 600°C. K, Ca, Mg, N, P, Zn, and Mn % increases by increasing pyrolysis temperature up to 2.3%, 1.2%, 0.8%, 0.6%, 0.18%, and 0.004% in case of LMBC and 2.2, 1.3, 0.4, 0.8, and 0.8% in case of PABC manufactured at 600°C. Significance of the data was estimated by *p* value <0.05. Fe in LMBC enhanced from 0.2 ± 0.1% at 400°C to 0.5 ± 0.2% at 600°C, while in PABC, it increased from 0.13 ± 0.2% to 0.39 ± 0.01%. K content elicited a significant improvement, from 0.7 ± 0.1% to 2.3 ± 0.1% in LMBC, and from 0.67 ± 0.15% to 2.2 ± 0.1% in PABC, across the elevating pyrolysis temperature range. Likewise, Ca uplifted from 0.3 ± 0.1% to 1.2 ± 0.1% in LMBC, and from 0.3 ± 0.1% to 1.3 ± 0.1% in PABC, and Mg elevated from 0.2 ± 0.1% to 0.8 ± 0.1% in LMBC, and from 0.2 ± 0.1% to 0.4 ± 0.1% in PABC. N was consistent with LMBC of 0.5–0.8 and PABC of 0.5–0.7. P in LMBC deviated between 0.12% and 0.20%, whereas in PABC, it remarkably increased from 0.2 ± 0.1% to 0.8 ± 0.1%. Zn rose from 0.0013% to 0.004% in LMBC and 0.002% to 0.006% in PABC, while Mn increased from 0.02% to 0.05% in LMBC and 0.02% to 0.08% in PABC, as pyrolysis temperature increased from 400°C to 600°C (Figure 6).

### 3.6. SEM of Biochar Samples

**Figure 7:**
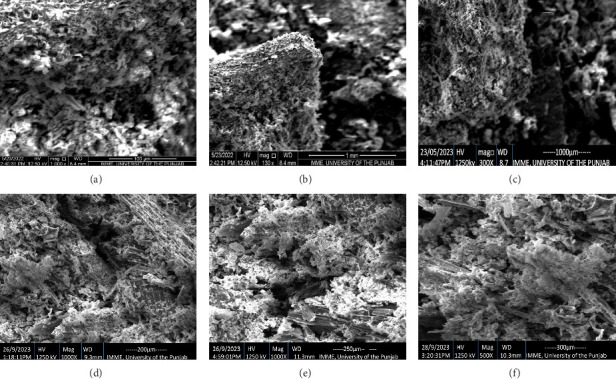
SEM of biochar samples. (a) LMBC 400. (b) LMBC 500. (c) LMBC 600. (d) PABC 400. (e) PABC 500. (f) PABC 600.

Morphology of LMBC and PABC was analyzed through SEM, which provided insights to understand the physical structure and surface (Figure 6). Images can be seen at high resolution to get a better idea of the study of the biochar surface. The current study reveals that the porosity increases as the pyrolysis temperature increases. Pyrolysis temperature modified the structure to a greater extent, as can be observed in the SEM image shown in Figure 7. LMBC and PABC made at 600°C showed maximum porosity and feathery structure, indicating the carbonaceous framework. Low temperature, i.e., 400°C produced macropores, while 500°C and 600°C gave rise to micropores, which are ideal sites for nutrient retention and microbe association.

### 3.7. XRD Pattern of Biochar Samples

**Figure 8:**
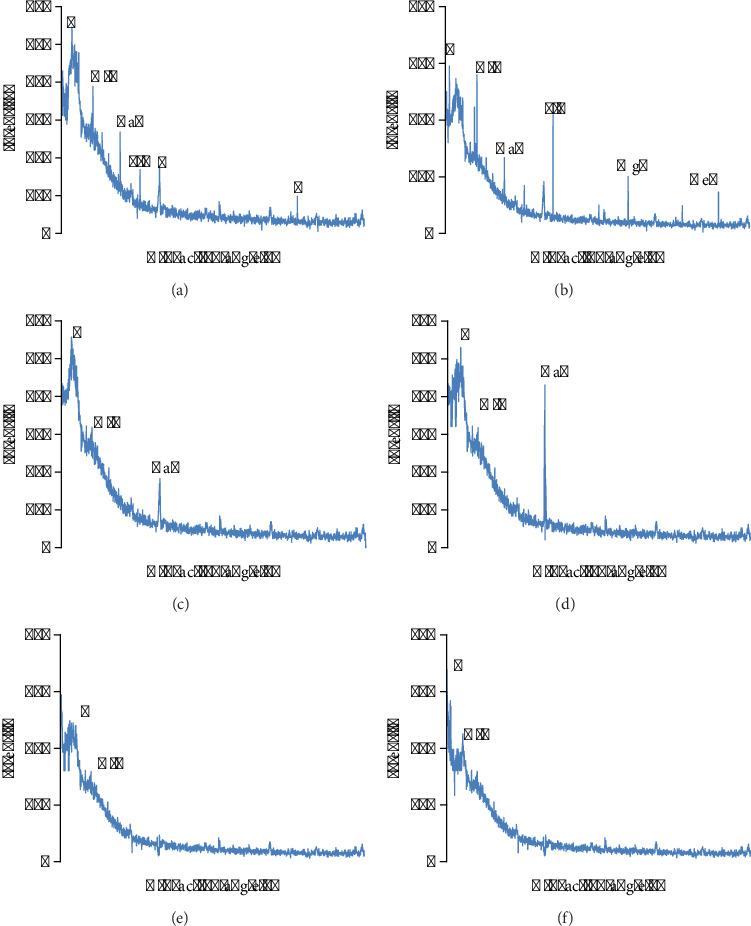
XRD pattern of biochar. (a) LMBC 400. (b) LMBC 500. (c) LMBC 600. (d) PABC 400. (e) PABC 500. (f) PABC 600.

Biochar samples were subjected to XRD to analyze the crystalline nature of biochar, which either is crystalline or amorphous after passing through various pyrolysis temperatures (Figure 8). Bragg's angle (2*θ*) was used to express the diffraction pattern. Some peaks were detected at 2*θ* value, indicating the element with some crystals, which reduces with respect to the pyrolysis temperature.

### 3.8. FTIR Analysis of Biochar Samples

**Figure 9:**
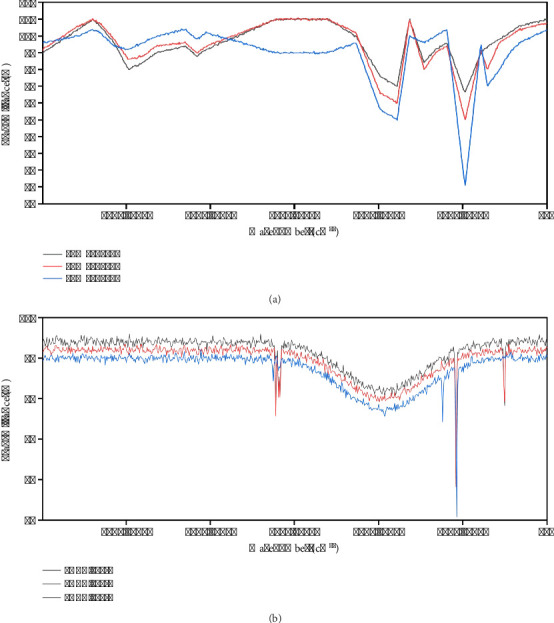
FTIR analysis of biochars. (a) LMBC 400, LMBC 500, and LMBC 600. (b) PABC 400, PABC 500, and PABC 600.

Pyrolysis temperature significantly affects the functional groups of biochar as represented in Figure 9. It is clear from the results that the functional groups are adherent to the biochar surface. Figure 8 indicates the complexity of changes in chemical bonds. Elevation in temperature imparts a lot of variations in biochar functional groups equally. The elevation of pyrolysis temperature (400°C–600°C) reduces the absorption intensities, stretching at the hydroxyl group (-OH) and carbonyl (C=O) vibration, respectively. The peaks between 2700 and 3500 cm^−1^ indicate the aliphatic (–CH) stretching, which lowers with respect to elevating temperatures. The -CH (1450–1570 cm^−1^) stretching indicates lignin and cellulose, while the -C-O-C-stretching indicates lignocellulose, which diminishes by increasing pyrolysis temperature, indicating the aromaticity of carbonaceous compounds.

### 3.9. Soil Analysis (Presowing and Postharvesting)

**Figure 10:**
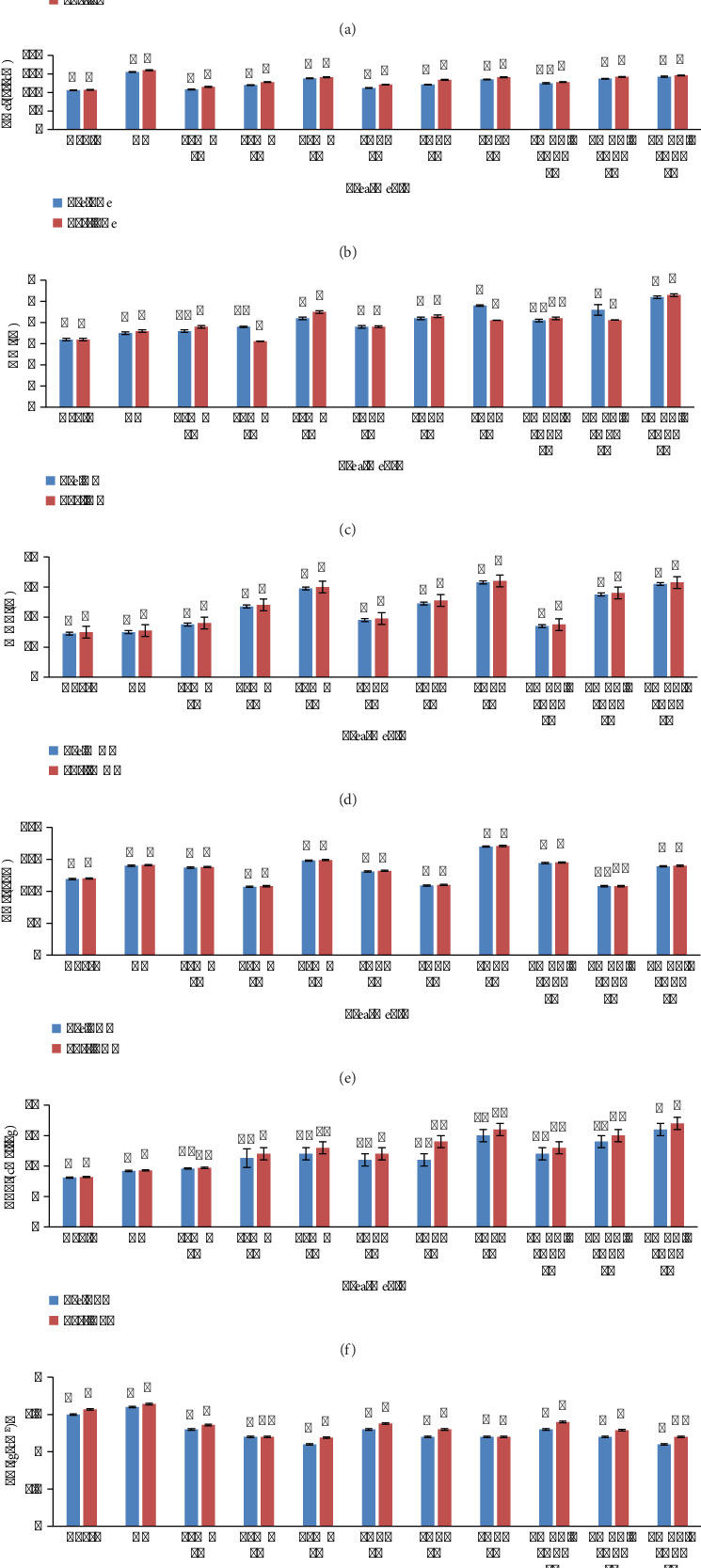
Soil physicochemical characterization of biochar amendment presowing and postharvesting. (a) pH. (b) ECe (dS/m). (c) OM (%). (d) WHC (%). (e) TDS (ppm). (f) CEC (cmol/kg). (g) BD (g/cm^3^).

Addition of biochar significantly improves the soil physical and chemical properties as shown in Figure 10, where statistical significance was <0.05. pH of BC-amended soil increases when amended with LMBC and PABC made at 600°C, i.e., 7.2, 8, 7.6, 8.1, 8.8, 7.8, 8.5, 8.6, 7.6, 8.6, and 8.8 for Cont, CF, 1% LMBC, 3% LMBC, 5% LMBC, 1% PABC, 3% PABC, 5% PABC, 1% LMBC + PABC, 3% LMBC + PABC, and 5% LMBC + PABC of presowing soil, and the pH of postharvested soil recorded 7.2, 8, 7.6, 8.1, 8.8, 7.8, 8.5, 8.6, 7.6, 8.6, and 8.8. ECe of BC-amended soil increases dramatically under the influence of biochars. The highest ECe 138 dS/m, 135 dS/m, and 142.3 μs/cm were monitored in soil amended with 5% LMBC, 5% PABC, and 5% LMBC + PABC, respectively, in presowing soil, likewise 141 μs/cm, 141 μs/cm, and 145 μs/cm were observed in postharvest. The OM% was ascertained at maximum 4.2%, 4.8%, and 5.2% in presowing soil and 4.5%, 4.1%, and 5.3% in postharvest soil when amended with 5% LMBC, 5% PABC, and 5% LMBC + PABC. WHC% of soil was seen to be the highest, 59%, 63% and 62%, when amended with 5% LMBC, 5% PABC, and 5% LMBC + PABC in presowing soil and similarly 60%, 64%, and 63% in postharvest soil. The TDS (ppm) was recorded as highest as 148 ppm, 170 ppm, and 139 ppm in presowing soil in 5% LMBC, 5% PABC, and 5% LMBC + PABC amendments and 149 ppm, 171 ppm, and 140 ppm in postharvest soil. The CEC (cmol/kg) was assessed maximum as 12 cmol/kg, 15 cmol/kg, and 16 cmol/kg in presowing soil in 5% LMBC, 5% PABC, and 5% LMBC + PABC amendments and 13 cmol/kg, 16 cmol/kg, and 17 cmol/kg in postharvest soil, respectively. The highest BD (g/cm^3^) was calculated as 1.18 (g/cm^3^), 1.2 (g/cm^3^) and 1.18 (g/cm^3^) in presowing soil and 1.19 (g/cm^3^), 1.21 (g/cm^3^) and 1.2 (g/cm^3^) in postharvest soil under the treatment of 5% LMBC, 5% PABC, and 5% LMBC + PABC.

### 3.10. Vegetable Productivity and Yield

**Figure 11:**
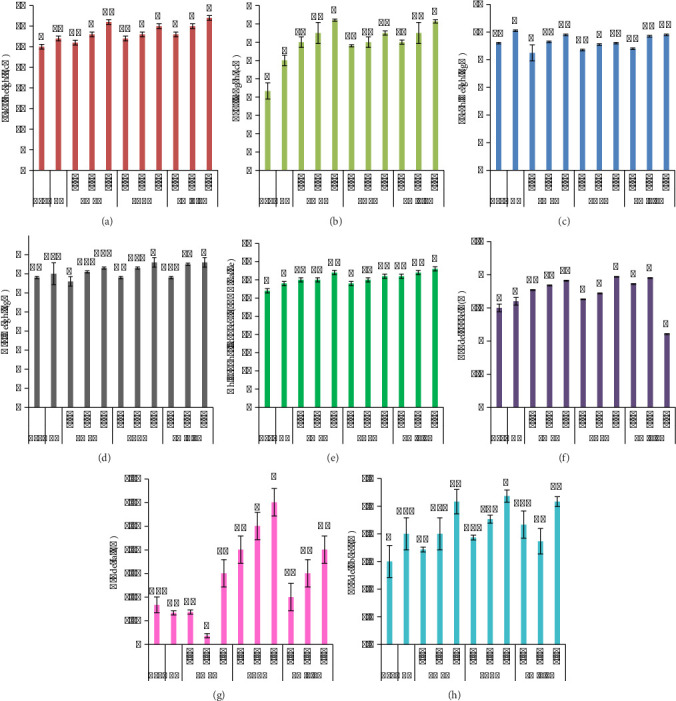
Spinach productivity and yield when grown in biochar amended soil. (a) Plant height. (b) Root length. (c) Fresh weight. (d) Dry weight. (e) Chlorophyll content. (f) Crude protein. (g) Crude fat. (h) Crude fibers.

Under the influence of biochar treatments, an excellent boost in the plant growth aspect was observed (45 days old plant), as shown in Figure 11 (*p* value <0.05). The highest length of shoots was seen in *Spinacia oleracea* L. when grown in soil mixed with 5% LMBC and 5% LM + PA biochar as compared to the Cont (36 cm and 37 cm, respectively). Root length was a maximum of 8.2 cm when mixed with 5% LMBC, then 8.1 cm in 5% LM + PA biochar amendment. Fresh weight of the plant was recorded as 9.8 g, which is the highest in both LMBC and LM + PA biochar when amended with 5% in the soil. The dry weight was calculated to be 7.6 g in PABC and LM + PA biochar 5% amendment as maximum. SPAD value of chlorophyll content was 38 in 5% LM + PA biochar, which is the highest among all treatments. The highest percentage of crude protein was recorded at 1.10 in 5% LM + PA biochar. The highest value of crude fat % was 0.06 in the LMBC 5% amendment. Crude fiber % was the highest, 3.83, in plants grown in 5% PABC amendment.

### 3.11. Correlation of Temperature (400, 500, and 600°C) of Pyrolysis With Biochar Properties

**Figure 12:**
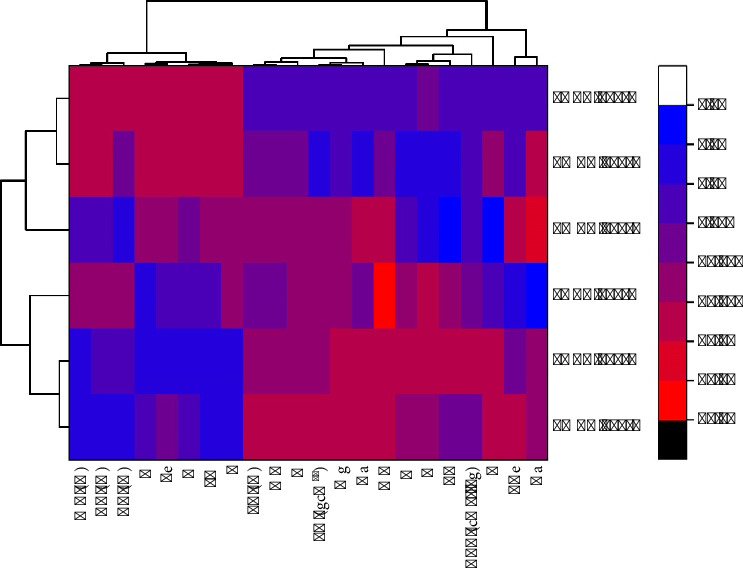
Heatmap with dendrogram showing the effect of pyrolysis temperature (400°C, 500°C, and 600°C) on biochar properties.

The heatmap in Figure 12 presents the effect of pyrolysis temperature, which is represented by 400°C, 500°C, and 600°C on physicochemical characteristics of biochar. As the temperature during pyrolysis rises, the carbon content (C), FC, and pH all rise, but the MC and VC decline. The change in elemental content is also seen where at higher temperatures, an increase in mineral-based elements such as Ca, Mg, and Mn) was observed along with an increase in BD and CEC. These results suggest that biochar produced with increased pyrolysis temperatures has a more stable carbon, lower volatile components, and altered mineral materials, which have implications for its soil improvement potential and carbon storage.

### 3.12. Correlation Between Concentrations of Biochar Treatments and Plant Growth and Yield

**Figure 13:**
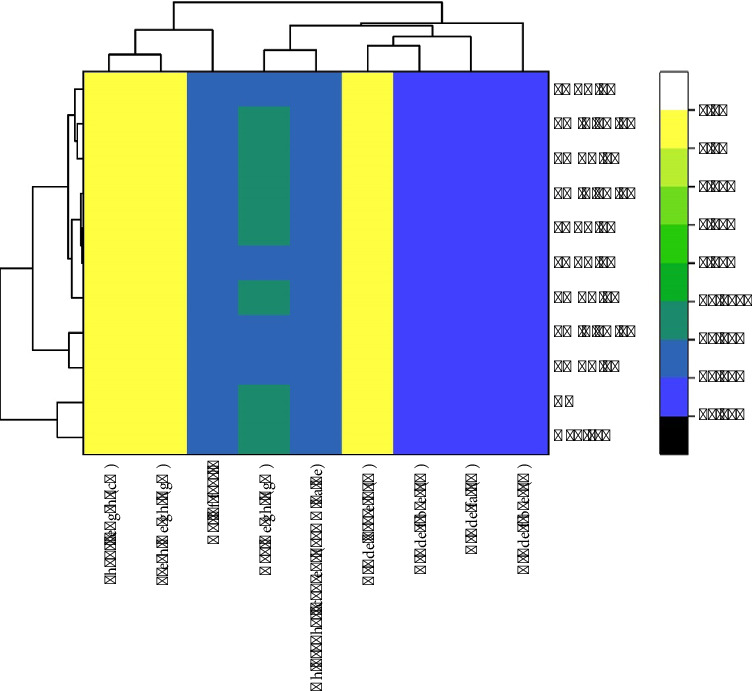
Heatmap with dendrogram showing the effect of different treatments on plant growth and yield.

The heatmap in Figure 13 shows the results of various treatments, PABC, LM + PA, LMBC, CF, and Cont, performed in various concentrations (1%, 3%, and 5%) on various plant variables, shoot length, fresh weight, number of fruits, dry weight, chlorophyll content (SPAD value), and crude components (protein, fiber, and fat). The top and left hierarchical clustering divides the treatments and variables by their similar patterns. The color gradient is high (*yellow*) to low (*dark blue*) and shows the observed values and differences among treatments. In this context, the positive effects are observed in PABC 5%, as well as LM + PA 5%, whereas the adverse effects are witnessed in PABC 1%, LMBC 1%, and CF. The results indicate that treatment effects on the growth of the plants vary, and some of them have a positive result, while others have a less positive result.

## 4. Discussion

Duckweed (*Lemna minor*) and common reed (*Phragmites australis*) are potent weeds that are massively found worldwide and also in Pakistan, where they are considered as useless or most of the time, harmful to the ecosystem, so they must be managed in a sustainable eco-friendly way. Biochar formation is one of the best strategies for managing these weeds which is a value-added product for the amelioration of soil. So, this study not only supports the use of LMBC and PABC as an effective soil ameliorant but also boosts the spinach yield, growth, and nutrient value in an excellent way.

LMBC and PABC were manufactured in a semiautomated charcolator under three different temperatures, i.e., 400, 500°C, and 600°C, as manufactured in our previous study [37]. By increasing the pyrolysis temperature, the yield decreases, as it is obvious that the increase in temperature leads to an elevation of the C content of LMBC and PABC and a decrease in O and H content [53]. This finding was aligned with the study of Dhinesh et al. and Xiao et al. [54, 55], in which they justified that the reduction in yield may happen due to evaporation of many volatile compounds. A decrease in biochar yield happens due to oxidation or volatilization of many compounds (Mašek et al. [56]). According to Titiladunayo et al. [57], the temperature, residency time, and heating rate affect the yield of biochar. The higher the temperature, the more industrial quality biochar will be produced, having high C, as shown in the proposed study [58]. High temperature transforms the biomass into carbon-rich material char as depicted by Nuwarapaksha et al. [59]. The produced biochars had the same results as reported by various research articles.

During combustion without oxygen, the lightweight compounds spaced out result in a high C/O ratio, as proved by the study of Naeem et al. [60]. Findings of the current study were supported by the study of Omotade et al. [61], in which it was proved that pyrolysis leads to thermal decomposition of biomass and H, N, O, and S get decreased due to various factors including volatilization, decomposition of functional groups, carbonization, and formation of gaseous compounds such as oxides of sulfur, N, or hydrogen. Increase in pyrolysis temperature leads to an elevation in C content of biochar and a decrease in O and H content [53].

MC of LMBC and PABC biochar decreases with elevating temperature. This may happen due to evaporation of moisture by applying heat, so the more we increase the temperature, the MC evaporates more [62]. MC may decrease due to thermal power and kinetic energy, which can break many compounds holding water, as proved by the study of Conz et al. [63]. Feedstock undergoes physical and chemical changes during pyrolysis, which results in lowering moisture [54]. This may happen due to high temperature, which releases the volatile compounds associated with chars, as presented by the study of Hossain et al. [64]. High temperature also bursts a significant amount of ash and FC as pyrolysis continues [58], as found by the proposed study in which LMBC and PABC produced more C content and AC with respect to increasing temperature. The reason behind this is that high temperature increase the volatility of many gases and liquids from biomass, leaving behind ash-forming minerals such as silica, Ca, and K [65].

pH of LMBC and PABC increases with respect to increasing pyrolysis temperature. From the previous studies on physical and chemical properties of biochar, it was obvious that the pH increases in proportion to temperature due to the decomposition of many acidic functional groups, leading to high alkalinity and ash formation. High temperature may retain many inorganic minerals, which increase pH by reducing acidity [66, 67]. High temperature makes LMBC and PABC more stable and porous, leading to an increase in CEC [68]. Some studies show similar results to those shown by Tag et al. [69], according to which, the high degree of carbonization increases the cations holding the functional groups. BD of LMBC and PABC decreases with respect to pyrolysis temperature, which was, according to several evidence, that an increase in temperature causes structural changes and high porosity [57]. For example, due to porous structure, the surface area increases and the BD decreases, making the char lightweighted and ideal [70]. Due to ash formation, structural and chemical modifications the increase in pyrolysis temp decreases BD [71]. In the current studies, similar results were seen as already proved by the researchers; they found that the BD always decreases with respect to manufacturing temperature. ECe of PABC and LMBC increases with increasing pyrolysis temperature. With reference to many studies conducted on biochar, it was seen that the ECe was affected by graphitization, which promotes the mobility of electrons and formation of graphite-like structure [72]. An increased porous structure also increases the space for trapping charges. Typically, high pyrolysis temperature leads to the formation of pure carbon which facilitates the conductivity and removal of functional groups [73].

Nutrient values of biochar increase with increasing temperature, as proved by experimental evidence. By increasing the temperature of pyrolysis, the nutrient content of biochar was elevated, which aligned with findings of previous literature. For example, the low pyrolysis temperature retains maximum nutrients but in restricted amounts because they are locked up chemically according to a study of Al-Wabel et al. [74]. At medium to high temperatures, the availability of nutrients increases due to the loss of extra compounds by evaporation. Although the high temperature yields carbon-rich and stable biochar [75], the concentration of N reduces, and the micronutrients increase by increasing the temperature of pyrolysis [76]. At low to medium temperature pyrolysis, the N content increases, but at high temperature, it fluctuates to a lower amount [77]. This may happen because of the volatilization of N [78]. A decrease of total N in the biochars occurred by removal of the ammonium-N and nitrate-N fractions at low to medium temperature [24]. However, high temperature (up to 600°C) N transformed into pyridine-like structures, the NH_4_-N and NO_3_-N, which have important agronomical characteristics because plants mostly uptake N from soil in the form of nitrates and ammonium ions [79]. All these research findings have supported the current experimentation and findings of analyses.

Biochars manufactured at 400°C, 500°C, and 600°C were different from each other in structure. For example, Das et al. [39] proved that apart from pyrolysis temperature, time of residence also influences the biochar's structure and properties; this is because the high temp caused the formation of excessive pores. At 400°C, the volatile compounds did not escape, and the structure seemed to be compact. The most fascinating thing is that the high pyrolysis temperature enhances the pore formation, augmenting the surface area, which ensures the charring procedure and volatilization [80]. At 400°C, the char contains maximum nutrients and minerals, but at 500°C and 600°C, the nutrients are released and can be detected maximally by EDX [81]. XRD analyses revealed that high temperature of pyrolysis leads to the formation of graphitic or carbonaceous structure as well as the destruction of many smaller peaks as shown in our study. For example, Adesemuyi et al. [82] described that high pyrolysis temperature reduces the peaks' sharpness as they decompose due to temperature. Similar findings were seen in the study of Muhammad et al. [83]. High pyrolysis temperature makes biochar more aromatic and carbonaceous as evidenced in FTIR analyses of LMBCs and PABCs. These results were incoherent with the study of Liu et al. [84] and our previous study [37]. Akter et al. [85], Mutolib et al. [86], and Munir et al. [37] described the same results in which the presence of aromatic compounds was reported in high pyrolysis temperature biochar.

By adding biochars, for soil fertility, the increased pH neutralizes the acidity of the soil [87]. Similar findings were ascertained by the proposed study of LMBCs and PABCs, in which the pH of the soil increased to an alkaline state. Basic cations facilitate the retention of nutrients and soil health [88], which is why biochar designed at high temperature had high CEC. Biochar increases the ECe of soil due to its porous morphology and soluble salts [25]; the same trend was observed in the case of LMBC and PABC. This increases nutrient retention, which ameliorates the soil and is beneficial for agronomic use [89]. Chars improve the SOC and sequester carbon for long-term storage [90] and promote better aeration and porosity, which is ideal for microbial activity [91]. An increase in SOC may result in a better texture of soil, which is necessary for plant growth, and it increases the water retention capacity of soil [92, 93]. High C in soil increases the carbon sequestration, improves soil structure and CEC, and improves water and nutrient retention of soil. By following the same trend as discussed in literature, the LMBC and PABC amendment in soil significantly increases the porosity and aeration, which hold water and nutrients. Study of Poveda et al. [94] also concluded that biochar amendments reduce leaching and increase soil health and affect soil structure and WHC, which lowers the leaching effect and concentration of TDSs. CEC of LMBC and PABC increases, which is necessary for soil health, as reported by Verma et al. [95]. Other researchers also derived the same results after assessing the physical and chemical properties of biochar-amended soil. For example, a high surface area has high adsorption for cations [96]. The availability of functional groups promotes the cation retention [97]. These cations are essential for plant growth, for instance, K, Ca, and Mg. The reduction in soil BD is due to biochar aeration, and the structure improves [98]. It may promote soil aggregation, which is necessary for root growth, retention of water, and availability of nutrients [99]. LMBC and PABC also reflected the matching trend in BD reduction. As spinach is a well-known and important vegetable, which is consumed by the worldwide population, it was selected as a test crop according to season and growth conditions. Under the influence of LMBC and PABC, it showed remarkable improvement in its growth and yield as well as its biochemical productivity. Similar results were observed by Jabborova et al. [100], who applied biochar for spinach growth. These consequences coincided with a study of Ahmad et al. [101] and Munir et al. [37], which revealed the potential biochar amendments that proved very effective for the growth of vegetables. The result of the experiment shows that the addition of biochar remarkably boosts the soil physicochemical characteristics, which leads to soil amelioration and an increase in the yield of vegetables. All the results were statistically significant. The use of biochar made of aquatic weed as a soil conditioner has great agronomic potential. Its large surface area and porous nature increase soil WHC, therefore making crops more resilient when facing water shortage. Besides, biochar is a storehouse of nutrients and enhances CEC that facilitates nutrient availability and decreases leaching loss. As a result, the biochar can be partially used instead of synthetic fertilizers, which will reduce input costs and reduce environmental pollution. Its stable carbon composition also helps in SOC accumulation and long-term fertility, which is sustainable in increasing vegetable productivity with less reliance on chemical fertilizers.

In the first heatmap with dendrogram in Figure 12, the effects of three temperatures of pyrolysis (400, 500°C, and 600°C) on the physicochemical composition of biochar were identified. A gradual increase in temperature is associated with an increase in C, FC, and pH, but a decrease in MC and VC. These empirical findings align with the literature [102, 103], showing that increasing the pyrolysis temperature has the effect of producing biochar with stabilized carbon and decreased volatile matter and increasing pH. The results are thus indicative of the fact that higher temperature–produced biochar can provide greater stability and higher long-term carbon sequestration capability. The heatmap also shows that the biochars produced with higher pyrolysis temperatures have greater concentrations of mineral elements (Ca, Mg, and Mn) and a greater BD and CEC. These qualities are mentioned as the ones that are necessary to enhance the quality of soil. Higher mineral content and CEC of biochar created at higher temperatures are therefore indicators of increased potential as soil amendments that may increase nutrient availability and improve soil structure. The correlation between temperatures of pyrolysis and properties of biochar shows the potential of using high-temperature biochars in soil-enhancement processes and long-term storage of carbon. Because such biochars have more stable forms of carbon and higher mineral levels, they can potentially perform the dual role of carbon sink and sustainable soil amendment to enhance fertility and structure [104]. The second heatmap in Figure 13 explores the results of five treatments (PABC, LM + PA, LMBC, CF, and Cont) at three concentrations (1%, 3%, and 5%) on the variables of plant growth. Hierarchical clustering shows that PABC 5% and LM + PA 5% treatments record positive results in shoot length, fresh weight, number of fruits, and chlorophyll content, as indicated by high values (yellow) in the heatmap. On the other hand, PABC 1%, LMBC 1%, and the CF treatment negatively impact these variables, as seen in their values presented by the lower intensity of the heatmap (dark blue). The beneficial effects of increasing PABC and LM + PA concentrations could be the improved nutrient uptake and physiological performance, as has been reported by Zhang et al. [105] that the growth-promoting compounds used at higher concentrations increase the chlorophyll content and biomass accumulation. In comparison, reduced PABC and LMBC concentrations with the addition of the CF treatment seem to inhibit vegetative growth because of the imbalances in nutrients or the toxicity impact. These findings support Lee et al. [106], who reported that the improper overuse of some treatments may cause nutrient stress that prevents plant growth. There is a relation between pyrolysis and plant growth; even though the two heatmaps reflect different experimental sectors, the results of the two heatmaps point in the same direction, trying to optimize agricultural inputs. Due to a higher level of carbon stability and better mineral content, biochars produced at high temperatures can enhance the health of soils and, therefore, increase the performance of the plants. Increased nutrient retention and pH adjustment also help in countering the negative responses of some of the growth treatments, especially in PABC 5% and LM + PA 5%, where growth responses have been registered. The initial results of this study were previously reported as a conference abstract at INCPS-2024 by Munir and Nazir, in which the potential of PABC and LMBC for soil amelioration and crop productivity was reported [35].

It is found that biochar may include toxic elements, i.e., heavy metals (e.g., Cd, Pb, and As), and their presence may present a risk unless properly characterized prior to use. However, aquatic weeds were collected from freshwater bodies where heavy metals cannot be found. Moreover, the rate of use is vital: low doses might not yield measurable effects, and overuse can cause alkalization of the soil or nutrient deficiencies or crop impairment. Moreover, the quality of biochar may be influenced by the variability in the feedstock type and pyrolysis conditions; thus, standardization and site-specific testing are required before its large-scale application can be adopted. Furthermore, this aquatic weed biochar can be used in future to test the productivity of other vegetables and crops, or it may be used for carbon sequestration studies and climate change mitigation, reduction of the fertilizer dependency, remediation of contaminated soil, integration with circular economy perspectives, tailored biochar for specific applications, and many other commercial applications.

Harvesting of aquatic weed biomass cannot raise significant environmental and ethical concerns. From an environmental point of view, it does not cause deforestation, loss of biodiversity, or degradation of soil, so this research work did not include any negative impact on the environment. Ethically and socially, this practice did not harm any local community but provided a sustainable harvesting solution for problematic aquatic weeds.

## 5. Conclusion

This research describes the valorization of two important and most prevailed aquatic weeds (common reed and duckweed) biomass by using oxygen-limited pyrolysis to form biochar. Biochar properties such as external surface features, effectiveness, and surface area vary with respect to varying temperatures of pyrolysis. Biochar pyrolyzed at 600°C and applied at 6% (w/w) concentration was declared as the most effective among all with respect to carbon sequestration, nutrient retention and release, and ameliorating properties. It efficiently ameliorated the soil chemical and physical properties, as well as wonderfully improved the yield, protein, carbohydrates, fats, and overall fiber content of the test vegetable spinach.

## Figures and Tables

**Table 1:** Nutrient analysis of biochar samples.

Nutrient analyses	LMBC			PABC		
	400°C	500°C	600°C	400°C	500°C	600°C
Fe	0.2 ± 0.1^ab^	0.4 ± 0.1^bc^	0.5 ± 0.2^c^	0.13 ± 0.2^a^	0.233 ± 0.01^ab^	0.39 ± 0.01^bc^
K	0.7 ± 0.1^a^	1.03 ± 0.01^b^	2.3 ± 0.1^d^	0.666667 ± 0.15^a^	1.5 ± 0.1^c^	2.2 ± 0.1^d^
Ca	0.3 ± 0.1^a^	0.7 ± 0.1^b^	1.2 ± 0.1^c^	0.3 ± 0.1^a^	0.7 ± 0.1^b^	1.3 ± 0.1^c^
Mg	0.2 ± 0.1^a^	0.4 ± 0.1^c^	0.8 ± 0.1^b^	0.2 ± 0.1^a^	0.2 ± 0.1^a^	0.4 ± 0.1^b^
N	0.5 ± 0.1^a^	0.8 ± 0.1^a^	0.6 ± 0.43^a^	0.5 ± 0.1^a^	0.7 ± 0.1^a^	0.6 ± 0.1^a^
P	0.2 ± 0.1^a^	0.12 ± 0.01^a^	0.18 ± 0.01^a^	0.2 ± 0.1^a^	0.8 ± 0.1^b^	0.8 ± 0.1^b^
Zn	0.0013 ± 0.01^a^	0.003 ± 0.01^ab^	0.004 ± 0.0^b^	0.002 ± 0.01^a^	0.004 ± 0.01^b^	0.006 ± 0.0^c^
Mn	0.02 ± 0.0005^a^	0.026667 ± 0.001^a^	0.05 ± 0.001^b^	0.02 ± 0.001^a^	0.02 ± 0.001^a^	0.08 ± 0.001^c^

*Note*. ± Standard deviations with a, b, and c letters showing the least significant difference (LSD) (*p* value < 0.05), *n* = 3.

## Data Availability

The data that support the findings of this study are available from the corresponding author upon reasonable request.
